# Supplementary material for: Superior Electrocatalytic Activity of MoS2-Graphene as Superlattice
Source: Nanomaterials (Basel). 2020 Apr 27;10(5):839. doi: 10.3390/nano10050839 (PMC7712152; doi:10.3390/nano10050839)
Supplement: Supplementary file 1 [file nanomaterials-10-00839-s001.pdf]

nanomaterials

Superior Electrocatalytic Activity of MoS_2-Graphene as Superlattice

Alejandra Rendón-Patiño [1], Antonio Domenech-Carbó [2], Ana Primo [1,*], and Hermenegildo García [1,*]

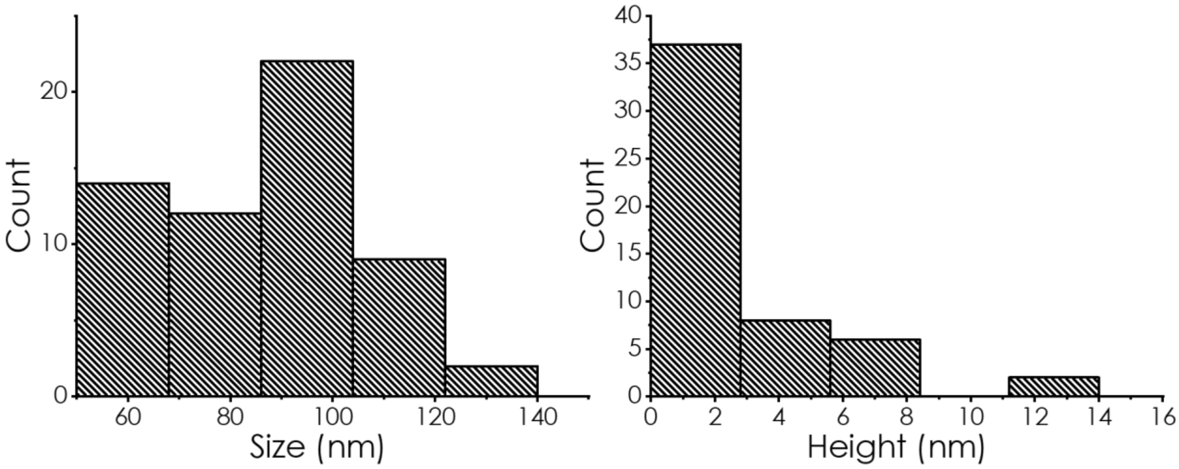

Figure S1. Lateral size (a) and height (b) of MoS_2 nanoparticles present on MoS_2/G.

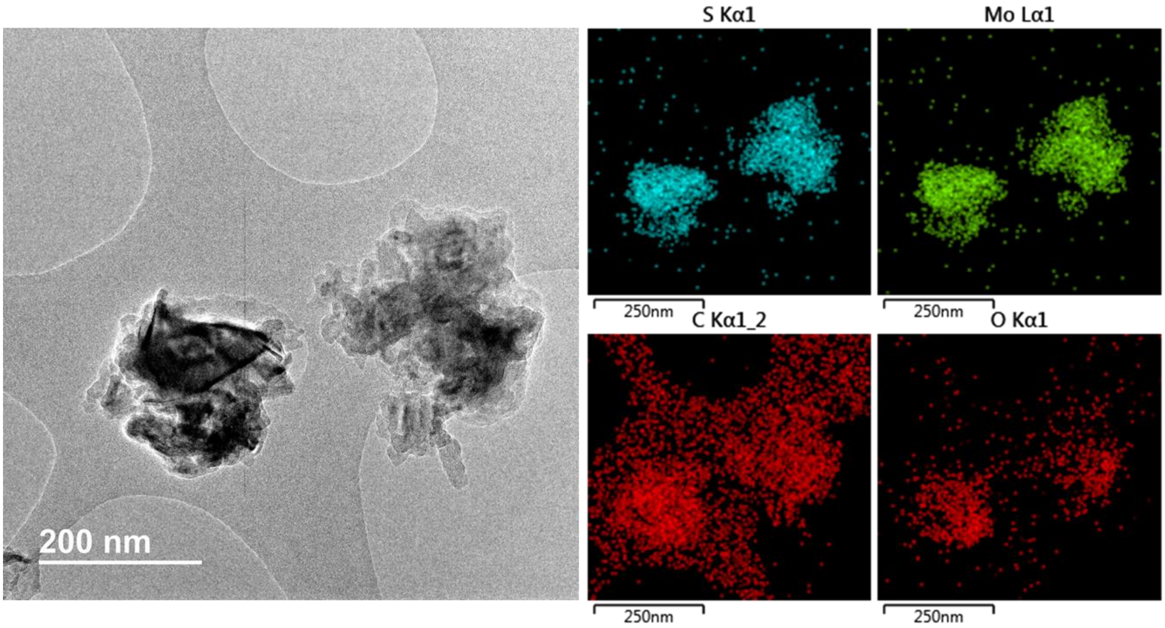

Figure S2. EDX analysis of S, Mo, C and O for the MoS_2/G particles shown in the TEM image (left frame).

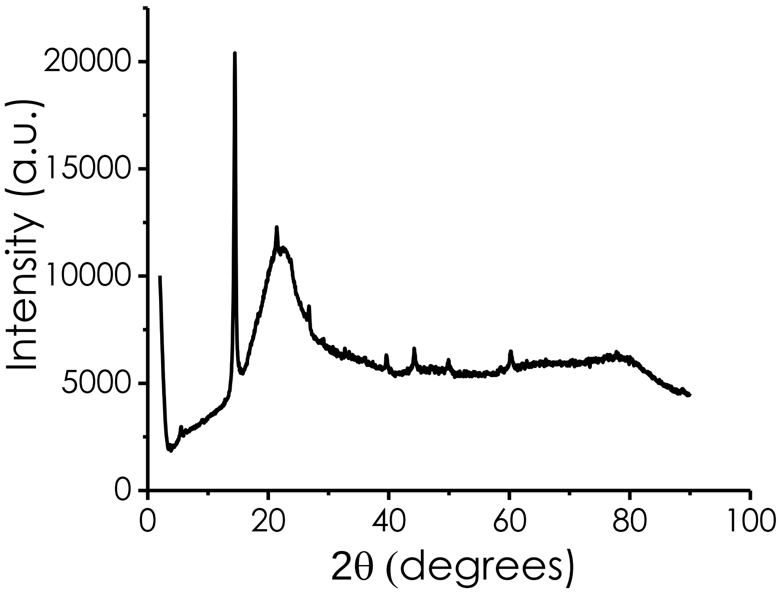

Figure S3. XRD spectrum of MoS_2/G.

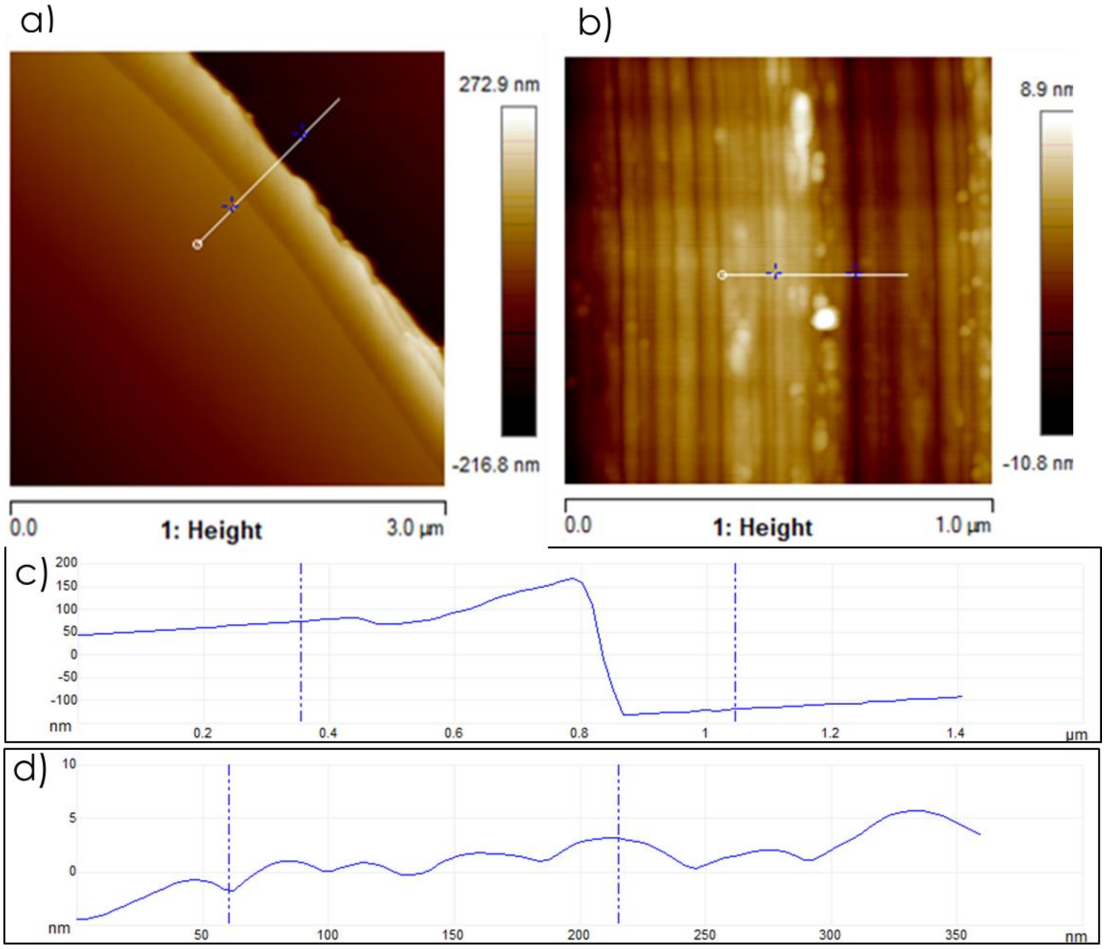

Figure S4. Frontal view (a and b) and height profiles (c and d) along the white lines of r-MoS_2/polystyrene (a and c) and r-MoS_2/G (b and d).

nanomaterials

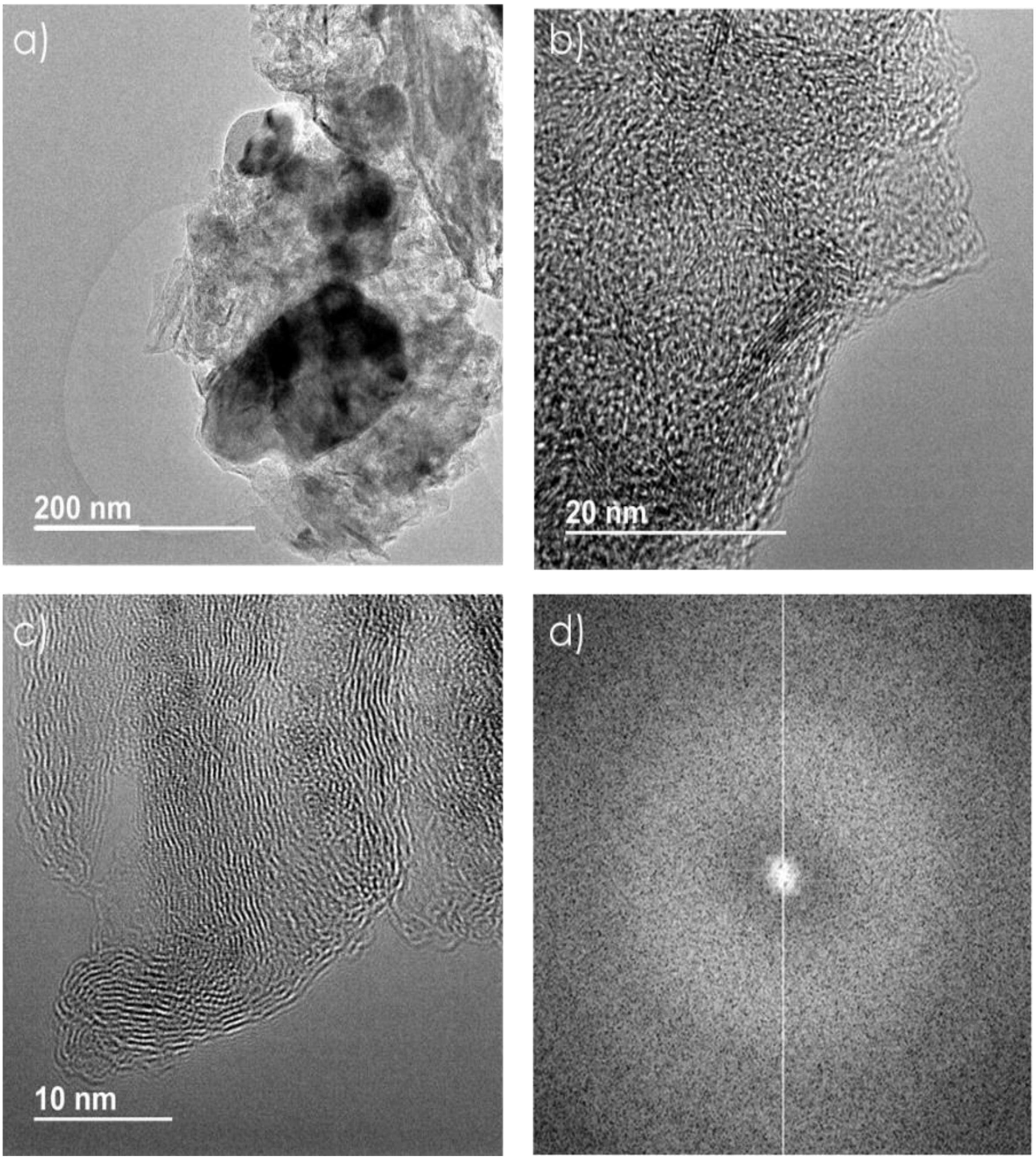

Figure S5. TEM at different magnifications and selected area electron diffraction of r-MoS_2/G.

nanomaterials

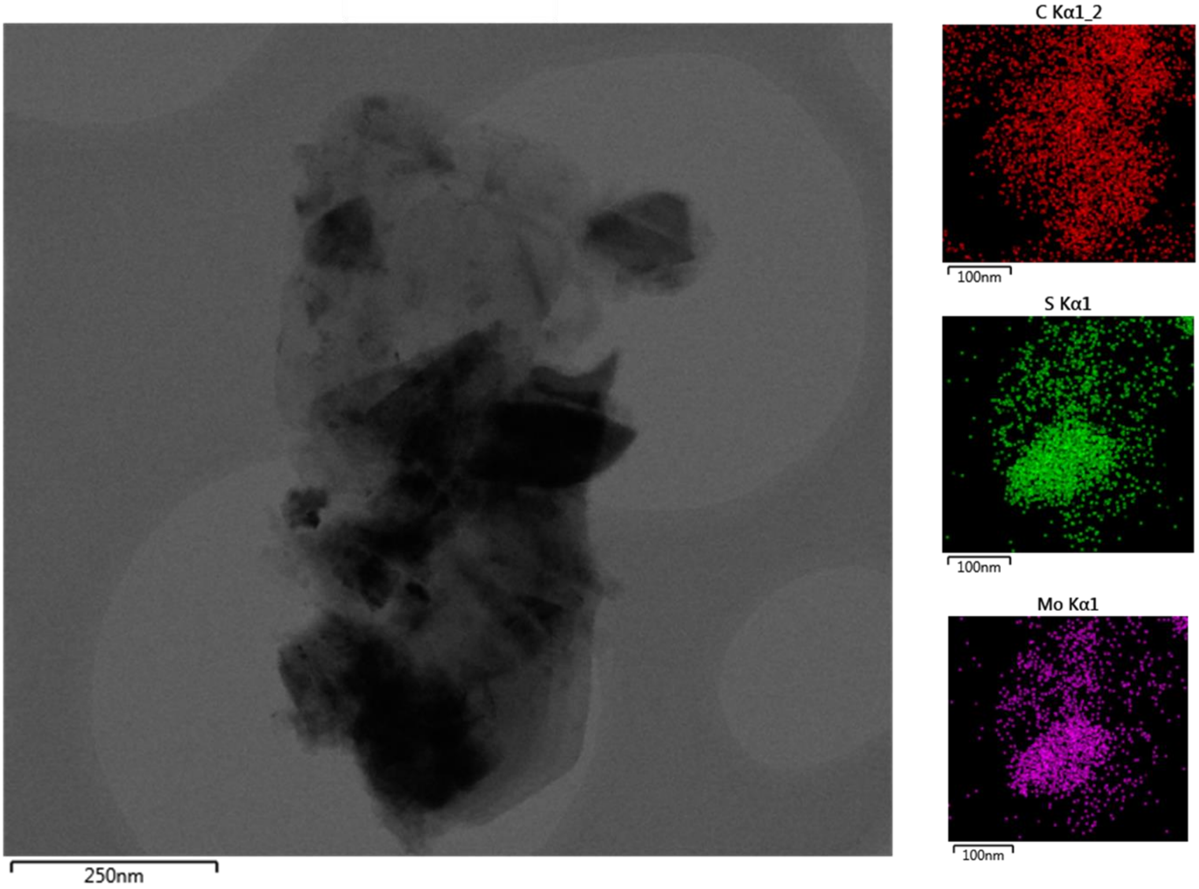

Figure S6. TEM image (left) and EDS analysis of C, S and Mo for r-MoS_2/G.
